# Different responses of alpine plants to nitrogen addition: effects on plant-plant interactions

**DOI:** 10.1038/srep38320

**Published:** 2016-12-06

**Authors:** Jun Wang, Peng Luo, Hao Yang, Chengxiang Mou, Li Mo

**Affiliations:** 1 Key Laboratory of Mountain Ecological Restoration and Bioresource Utilization & Ecological Restoration Biodiversity Conservation Key Laboratory of Sichuan Province, Chengdu Institute of Biology, Chinese Academy of Sciences, Chengdu, 610041, China; 2 College of Life Sciences, Sichuan University, Chengdu, 610064, China; 3 University of Chinese Academy of Sciences, Beijing, 100000, China

## Abstract

The different responses of plant species to resource stress are keys to understand the dynamics of plant community in a changing environment. To test the hypothesis that nitrogen (N) increase would benefit N competitive species, rather than N stress-tolerant species, to compete with neighbours, we conducted an experiment with neighbour removal, N addition and soil moisture as treatments in an alpine grassland on the southeastern Tibetan Plateau. Both growths and competitive-response abilities (CRA, the ability to tolerate the inhibitory effects of neighbors) of *Kobresia macrantha, Polygonum viviparum and Potentilla anserine* in wet site were facilitated by N addition, conversely, both growths and CRA of *Taraxacum mongolicum* and *Ligularia virgaurea* were suppressed by N addition, indicating that the responses of CRA of target species under N addition were consistent with the N utilization strategies of them. Moreover, the facilitative effects of N addition on competitive-response abilities of *Kobresia macrantha* and *Polygonum viviparum* were not found at the dry site, illustrating that soil moisture can alter the changes of neighbour effects caused by N addition. Life strategy of dominant species in plant community on the undisturbed southeastern Tibetan Plateau may shift from N stress-tolerant to N competitive, if the N increases continuously.

Both competition (negative effect) and facilitation (positive effect) between plants co-occur in stress environments^1^,^2^,^3^, and the effects of environmental changes on plant-plant interactions are among of the main mechanisms through which abiotic factors control plant community structure and ecosystem processes^4^,^5^. Many studies from both high and low altitudes have demonstrated that the net plant-plant interaction (the sum of the negative and positive effects), would shift from facilitative to competitive, along a gradient of alleviated environmental stress^6^,^7^,^8^, as predicted by the “stress gradient hypothesis (SGH)”^9^. Nevertheless, some studies from both alpine and arid regions found that the facilitative effects of neighbours either remained unchanged^10^ or increased with reduced abiotic stress^11^,^12^. The questions of if and how the species differentiate themselves in response to environment changes remain unclear.

Species differ in their ‘competitiveness’, ‘stress-tolerance’ (life strategies, following the CSR classification of Grime)^13^, ecological niches, and thus distributional optimum^14^,^15^, even in the same habitat. Therefore, species with different life strategies may also differ in their response to environmental change induced by climate change or shifts in vegetation structure, and their interactions with neighbours may change in different directions when abiotic factors are changed^16^,^17^. However, many previous studies on plant-plant interactions in response to stress were carried out in terms of species^2^,^3^,^7^,^10^,^12^, rather than in terms of life strategies of species. Maestre *et al*. found that the life strategies of studied species were among the key factors that affecting plant-plant interactions in response to changed stress^16^. He predicted that, in the case of resource (e.g., water, light and nitrogen) stress, only if the beneficiary (‘facilitated’ species)^16^ was stress-tolerant and the benefactor (‘facilitator’ species)^16^ was competitive would the net neighbour effects (tested by comparing performance of target individuals grown with and without neighbour vegetation) shift from facilitative to competitive under reduced stress, however, the response would be different under other conditions^16^. The role of life strategy in effecting the change of plant-plant interaction under reduced stress has been verified by Maestre *et al*.^18^ under the scenario of reduced water stress in a semi-arid Mediterranean steppe, and by Rebele *et al*.^19^ under the scenario of reduced nutrient stress on a glacial till plateau. Both of Maestre *et al*.^18^ and Rebele *et al*.^19^ found that when both the beneficiary and benefactor were stress-tolerant, the net effect of neighbour vegetation on beneficiary were positive under moderate stress (i.e., water/nutrient stress), but were negative under both high and low stress. Nevertheless, Liancourt *et al*.^14^ suggested that life strategies of species might not change the response of plant–plant interaction under reduced water stress in a meso-xeric calcareous grassland. Thus, it is not yet clear how plant life strategy will affect the balance between positive and negative interactions when environmental stress reduced, particularly in alpine ecosystems where is usually limited by nitrogen^20^,^21^.

Here we aim at testing the role of life strategy, plant-plant interaction and N addition for the diversity of alpine grassland on the Tibetan Plateau where is limited by N due to the low temperature and low decomposition rate^20^,^22^. In recent decades, an increase in N due to N deposition^23^, fertilization and accelerated mineralization^24^,^25^ has been recognized as one of the major drivers of vegetation change in the area^26^,^27^. Previous studies carried out on the Tibetan Plateau found that the effect of N increase on dominance of species is species specific^22^, nevertheless, most of the efforts that analyzing the reasons of species specific response have been paid to the response of the plant itself rather than to the response of plant-plant interaction. It would be interesting to explore how the changes in plant-plant interactions have contributed to the response of plant growth and thus vegetation changes under N addition.

To examine the effect of plant life strategy on plant-plant interactions in response to reduced N stress, we conducted a neighbour removal experiment with multiple species with different plant life strategies under N manipulation (simulation of nitrogen fertilizer) in a natural alpine grassland. Additionally, given that variations in soil moisture due to precipitation variation and human activities (e.g., artificial drainage or irrigation) also commonly drive vegetation changes on the Tibetan Plateau^28^, to test whether the effects of increased N on plant-plant interactions would be changed by soil moisture, we repeated the experiment at a nearby site where the soil moisture was significantly lower due to artificial drainage. We predicted that 1) the N increase would benefit N competitive species to compete with neighbours but would increase neighbour competition effects on N stress-tolerant species, and 2) the effects of N addition on plant-plant interactions would be altered by habitat soil moisture. We anticipate that the findings will help in understanding the dynamics of the plant community under complex climate change.

## Results

### Effect of neighbour removal on soil temperature

**Figure 1:**
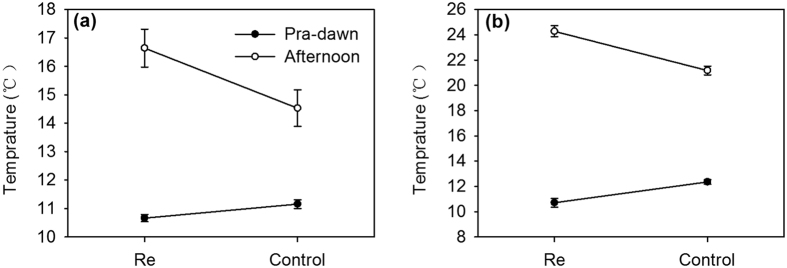
Effect of neighbour removal on soil temperature in the neighbouring area at 5 cm depth. (**a**) Mean (±SE) at the dry site and (**b**) wet site.

The removal of neighbours had significant effects on soil temperature in both sites, and the effects of neighbour removal on soil temperature in dry site were significant stronger than the effects in wet site (Fig. 1, Supplementary Table S1, in all cases, P < 0.05). Removal of neighbour decreased the pre-dawn soil temperature of the neighbouring area at the wet site and dry site by average values of approximately 0.49 °C and 1.65 °C, and increased afternoon soil temperature by average values of approximately 2.11 °C and 3.11 °C, respectively.

### Effect of neighbour removal on plant growth

**Figure 2:**
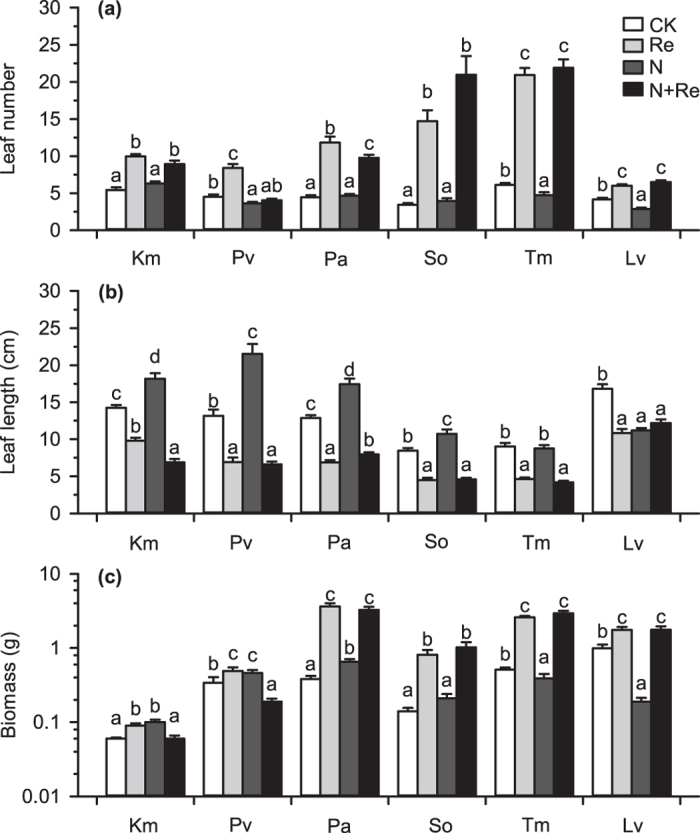
Plant growth response to neighbour removal and N addition. (**a**) Mean (±SE) of leaf number, (**b**) leaf length and (**c**) aboveground biomass of the target species (*Kobresia macrantha* (Km), *Polygonum viviparum* (Pv), *Potentilla anserine* (Pa), *Sanguisorba officinalis* (So), *Taraxacum mongolicum* (Tm) and *Ligularia virgaurea* (Lv)) at wet site with neighbours retained under natural conditions (CK), with neighbours removed under natural conditions (Re), with neighbours retained under N addition (N) and with neighbours removed under N addition (N + Re). The vertical axis of subfigure (**c**) was log-transformed to exhibit the difference of biomass among treatments.

As shown in the split-plot ANOVA tables (Supplementary Table S2) for the effects of year of experiment (Year), N addition and removal of neighbour (Re) on the growth of species at the wet site, there were no significantly interaction between Year and all combinations of N addition and Re for growth of all species (Supplementary Table S2, in all cases, P > 0.05), except for aboveground biomass of *T. mongolicum* under interaction between Year and Re (P = 0.007). The interaction between N addition and Re was significant for the growth of all species (Supplementary Table S2, in all cases, P < 0.05), except for all indexes of *S. officinalis* (Supplementary Table S2, in all cases, P > 0.05) and leaf length of *T. mongolicum* (P = 0.548). Without N addition and after neighbour removal, the leaf length of all target species was clearly reduced (Fig. 2b), and the leaf number was substantially increased (Fig. 2a), resulted in a marked increase in the aboveground biomass of these species (Fig. 2c).

### Effect of N addition on plant growth — evaluation of plant life strategy

**Figure 3:**
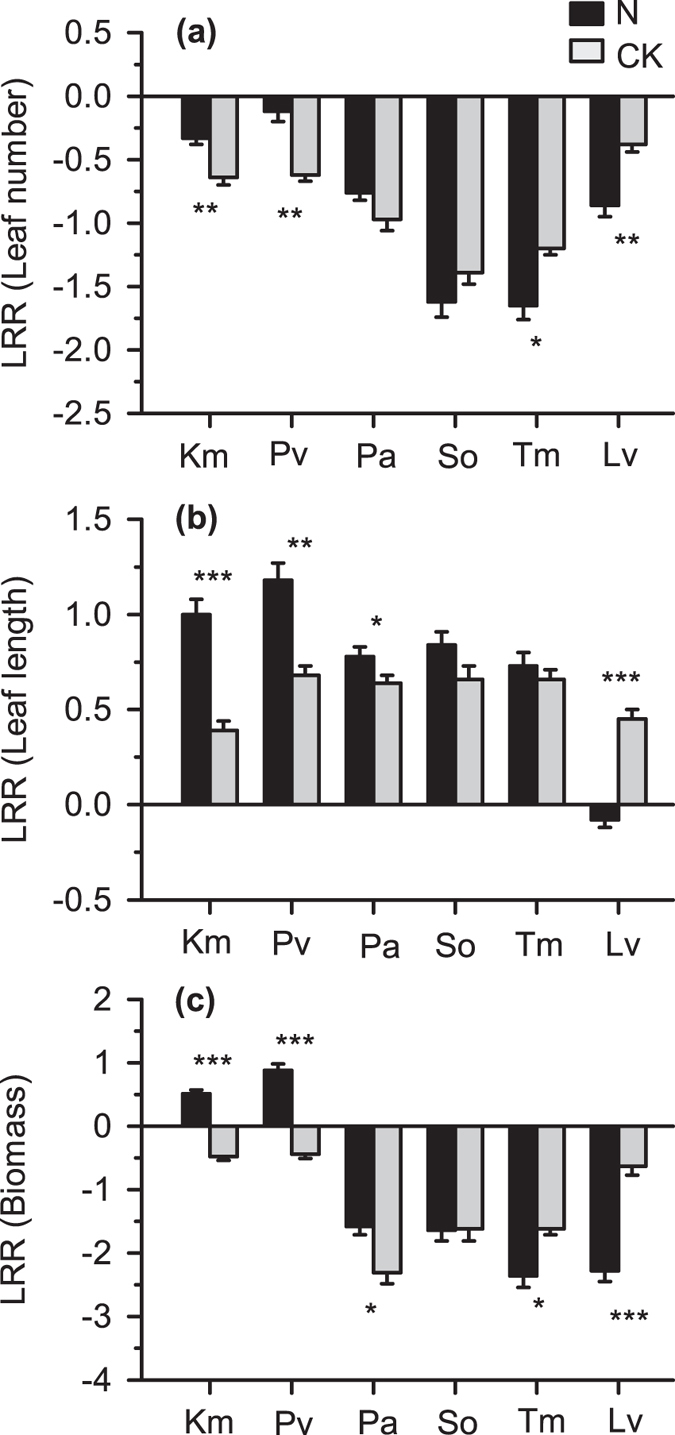
Mean log response ratio (LRR) of (**a**) Leaf number, (**b**) Leaf length and (**c**) Aboveground biomass of all target species (see Fig. 2 for the full name of each species) either receiving nutrients (N; black column) or not (CK; grey column) at wet site. Error bars represent 1SE. Asterisks above bars indicate the significance of difference between CK and N in a two-sample t-test: *P < 0.05; **P < 0.01; ***P < 0.001.

As shown in Fig. 2, when neighbours were retained, N addition had no substantial impact on leaf length of *T. mongolicum*, but obviously decreased the leaf number and the aboveground biomass of it by 22.5% and 23.5%, respectively (Fig. 2a,b). After N addition was applied, the leaf length and leaf number of *L. virgaurea* grown with neighbours were significantly reduced by 31.4% and 33.6%, respectively (Fig. 2a,b), which resulted in sharply decreased in the biomass of *L. virgaurea* by 80.8% (Fig. 2c). N addition significantly increased the leaf length of *S. officinalis* grown with neighbours by 27.0%, but did not significantly change the leaf number and aboveground biomass of this species. In contrast to *L. virgaurea*, *T. mongolicum* and *S. officinalis*, when neighbours were kept intact, N addition resulted in an obvious increase in the leaf length of *K. macrantha*, *P. viviparum* and *P. anserine* by 27.6%, 63.4% and 35.5%, respectively (Fig. 2b), but had no significant effect on their leaf number except for a significant but small decrease in the leaf number of *P. viviparum* (Fig. 2a), which led to a remarkable increase in aboveground biomass of them by 66.7%, 35.3% and 71.1%, respectively (Fig. 2c). When neighbours were removed, N addition reduced the leaf length of *K. macrantha* by 29.5% and the leaf number of *P. viviparum* by 51.8% (Fig. 2a,b), and thus decreased the biomass of *K. macrantha* and *P. viviparum* by 31.3% and 61.2%, respectively (Fig. 2c). In contrast to *K. macrantha* and *P. viviparum*, the growth of the other four species, except aboveground biomass of *S. officinalis*, was not affected by N addition on the bare land (Figs 2 and 3). The aboveground biomass of *S. officinalis* was increased by N addition on the bare land (Fig. 3c).

### Effect of N addition on plant-plant interactions

The results of split-plot ANOVAs for the effect of N addition and Year on neighbour effects on the leaf number, leaf length and aboveground biomass of each species are summarized in Supplementary Table S3. The results showed that there was no significant interaction between N addition and Year (Supplementary Table S3, in all cases, P > 0.05), which indicated that the year of experiment had no impact on the effects of N addition. N addition significantly reduced the negative effects of neighbours on the leaf number of *K. macrantha* and *P. viviparum* (Fig. 3a, for *K. macrantha*, P = 0.007; and for *P. viviparum*, P = 0.004), strongly reduced the negative effects of neighbours on the leaf number of *P. anserine* (Fig. 3a, P = 0.198), and obviously increased the positive effects of neighbours on the leaf length of these species (Fig. 3b, for *K. macrantha*, P < 0.001; for *P. viviparum,* P = 0.002; and for *P. anserine*, P = 0.014). In contrast, the negative effects of neighbours on the leaf number of *L. virgaurea* and *T. mongolicum* were significantly enhanced by N addition (Fig. 3a, *L. virgaurea*, P = 0.002; *T. mongolicum*, P = 0.045), and the net effect of neighbours on the leaf length of *L. virgaurea* shifted from positive to negative under N addition (Fig. 3b, P < 0.001). As a result, N addition sharply reduced the negative effects of neighbours on the aboveground biomass of *K. macrantha*, *P. viviparum* and *P. anserine* (Fig. 3c, for *K. macrantha* and *P. viviparum*, P < 0.001; and for *P. anserine*, P = 0.025), but obviously increased the negative effects of neighbours on the biomass of *L. virgaurea* and *T. mongolicum* (Fig. 3c, for *L. virgaurea,* P < 0.001; and for *T. mongolicum,* P = 0.048). N addition did not obviously change the effects of neighbours on the growth of *S. officinalis* (Fig. 3c, Supplementary Table S3, in all cases, P > 0.05).

### Interaction between soil moisture and N addition

As shown in the split-plot ANOVA tables (Supplementary Table S4) for the effects of Year, N addition and experiment site on the impacts of neighbours on number of leaves (LRR (LN)), leaf length (LRR (LL)) and aboveground biomass (LRR (AB)) of *K. macrantha* and *P. viviparum*, there was no significant interaction between Year and all combinations of N addition and experiment site, which show that the year of experiment have no obvious effect on the impacts of N addition and experiment site. The interaction effect between N addition and soil moisture was significant with respect to the neighbour effects on *K. macrantha* and *P. viviparum* (Supplementary Table S4, for LRR (LL) and LRR (AB) of *K. macrantha* and LRR (LN) and LRR (AB) of *P. viviparum*, P < 0.001; for LRR (LN) of *K. macrantha*, P = 0.001; and for LRR (LL) of *P. viviparum*, P = 0.024).

**Figure 4:**
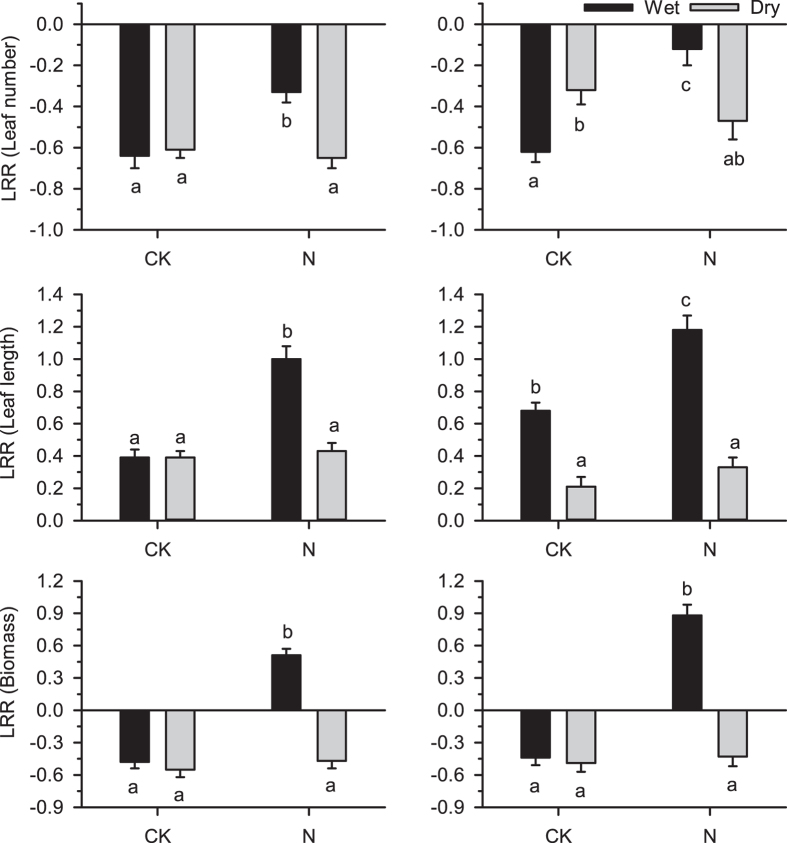
Mean log response ratio (LRR) of (**a,b**) Leaf number, (**c,d**) Leaf length and (**e,f**) Aboveground biomass of (**a,c,e**) *K. macrantha* and (**b,d,f**) *P. viviparum* either receiving nutrients (N) or not (CK) at dry site (Dry) and wet site (Wet). Error bars represent 1SE.

At the wet site, N addition not only significantly reduced the negative effects of neighbours on the biomass and leaf number of *K. macrantha* and *P. viviparum*, but also obviously enhanced the positive effect of neighbours on the leaf length of these two species, however, N addition had no obvious effect on the plant-plant interactions at the dry site (Fig. 4).

## Discussion

### Effect of neighbours on target individuals

Previous studies in high altitude grasslands have found that both competition and facilitation between individual plants in alpine habitats are common due to the multiple microclimatic effects provided by neighbouring vegetation^3^,^15^,^29^. In addition, individual tillers may change their internal resource allocation in response to neighbour removal^2^,^3^,^6^,^29^. In line with these studies^2^,^3^,^6^,^15^,^29^, we found that the leaf length of all target species was reduced when neighbours were removed, which implies a positive facilitative shelter effect of the neighbours against severe climatic conditions such as high solar radiation, strong wind and low temperature^3^. Our result reported a lower night soil temperature when neighbours were removed, which increased the risk of freezing injury. Additionally, the leaf number of the selected species was increased when neighbours were removed, showing a negative shading effect of neighbours on the growth of leaf buds^30^ and a competition effect for soil nutrients^2^,^3^,^29^. The growth of the leaf bud is sensitive to light intensity^30^, but shading may be considerable at high altitude due to frequent cloudy conditions^31^. From the perspective of internal resource allocation of target species, when there were neighbours around, due to shading of neighbours, they needed to invest more resources to grow longer (bigger) leaves to capture more light, while when neighbours were removed, they could invest the resources to grow more leaves rather than growing longer (bigger) leaves. Although competition and facilitation coexisted, neighbour removal increased the aboveground biomass of all target species, showing that the effect of competition was stronger than facilitation in controlling the aboveground growth of alpine plants.

### Evaluation of plant life strategy

Our results showed that the species we studied responded to N addition differently, reflecting distinct N utilization strategies. N addition increased the leaf length and the biomass of *K. macrantha*, *P. viviparum* and *P. anserine* at the wet site when neighbours were retained, indicating that these three species would be N limited in the studied communities and all of them should be N competitive. This is in line with other researchers, who found that the dominance of some sedge species in subalpine^32^ and alpine grassland^33^ and *P. viviparum* at polar semi-desert site^34^ was increased by N addition. Moreover, the positive effect of N addition on *P. anserine* might also be supported by the increased dominance of *P. anserine* in abandoned livestock enclosures (brush-ringed or fenced paddocks), where the soil is much richer in N content^35^. Bin *et al*. found that *Kobresia myosuroides* and *Potentilla fragarioides* were N limited in the alpine meadow on the Tibetan Plateau^36^, which could provide an indirect evidence to the N competitive of *K. macrantha* and *P. anserine*. In contrast to these N competitive species, the leaf number and length and the biomass of *L. virgaurea* and the leaf number and the biomass of *T. mongolicum* were reduced by N addition, suggesting that these species may not be N limited and may be N stress tolerant. Shi *et al*. found that *L. virgaurea* appeared mostly in extremely degraded grassland and could increase the N mineralization and N concentration of top soil in the alpine meadow on the Tibetan Plateau^37^, which support our result that *L. virgaurea* is N stress tolerant. The growth of *S. officinalis* was not affected by N addition (except its leaf length was elongated by increased N when neighbours were retained), implying that N might not be the main limiting factor for this species in our experimental site, and this species could be classified as having intermediate N sensitivity.

Interestingly, the growth of *K. macrantha* and *P. viviparum* was significantly negatively affected by N addition when neighbours were removed, suggesting that other neighbour facilitative effects existed in addition to the “shelter” effects related to severe climate. Previous studies showed that cascading effects (e.g., effects of animal and microorganism on plant) were crucial for plant growth^38^,^39^,^40^,^41^,^42^,^43^,^44^ and were sensitive to nitrogen^44^. In this study, vegetation might weaken the negative effects of N on the cascading effects by absorbing the added N and reducing soil N concentration^6^, implying that neighbours could also serve as a “refuge” from the negative effects caused by N addition. Our experimental design and measurements do not allow us to fully elucidate how the cascading effects were effected by N addition, a further study that takes the activities of soil animal and microorganism into consideration may provide us with more information.

### The effects of reduced resource stress on competitive response ability of plant

Revealing how plant-plant interactions, especially competitive response ability (i.e., the ability to tolerate the inhibitory effects of neighbours) change in response to reduced N stress will help us to understand the mechanism of changes in plant community composition and structure under N addition. The SGH model proposed that increased productivity due to N addition (reduced stress) would enhance the competition between plant individuals with respect to other resources such as light and space^9^, as a general response of plant-plant interactions to changes in stress. Nevertheless, many evidences have shown that the effect of reduced stress on plant-plant interactions may be species specific^10^,^11^,^12^,^17^,^45^. Cavieres *et al*. found that the neighbour facilitation effect on *Hordeum comosum* did not weaken under reduced cold stress at high altitudes in the Andes^10^, which did not fit the SGH model. Furthermore, Holzapfel *et al*.^45^ and Tielbörger *et al*.^12^ obtained opposite results under reduced water stress with different target species under similar conditions. In our experiment, when N addition was applied, the negative effects of neighbours on *L. virgaurea* and *T. mongolicum* (N stress tolerant) were enhanced, but the net effects of neighbours on *K. macrantha* and *P. viviparum* (N competitive) shifted from negative to positive and the negative effects of neighbour on *P. anserine* (N competitive) were reduced. Additionally, neighbour effects on *S. officinalis* (intermediate N sensitivity) did not change. These findings supported our prediction that the effect of increased N on competitive response ability of the target species is consistent with the N utilization strategies of it (e.g., N competitive vs. N stress-tolerant strategies). Furthermore, as Fu *et al*. found that N addition significantly and strongly increased aboveground biomass of graminoid, sedge and total community on the Tibetan Plateau^22^, the neighbouring species (mainly including graminoid and sedge) could be treated as N competitive strategy. Thus our study supported the prediction of Maestre *et al*. that the net neighbour (competitive strategy species) effect on competitive species will shift from competition to facilitation when the resource stress is reduced from high to medium^16^. Our study of N manipulation, together with Maestre *et al*.’s research along a gradient of precipitation in semi-arid Mediterranean steppes^18^ and Rebele *et al*.’s research along a gradient of nutrient in greenhouse^19^, illustrated the statement of Maestre *et al*., i.e., that the importance of plant life strategy should be fully taken into consideration in explaining and predicting the effects of environmental changes on plant-plant interactions and thus the dynamics of community structure and ecosystem functions^16^.

The different responses of the plant-plant interactions to N addition were a result of resource (e.g., light, water, space) reallocation induced by distinct N utilization strategies. When N addition was applied, although the neighbour vegetation was also facilitated by N addition as found by Fu *et al*.^22^, the N competitive species may have benefitted more from the extra N and grew faster than the neighbours; thus, the neighbour competition on other resources (e.g., light, space, water) should be relieved, such as in the cases of *K. macrantha*, *P. viviparum* and *P. anserine*. Additionally, the facilitative “refuge” effect of neighbours on *K. macrantha* and *P. viviparum* might also help them to grow considerably better in a natural community than on bare land. In contrast, although the growth of *L. virgaurea* and *T. mongolicum* on bare land were not changed by N addition, these species benefited less from N addition than the neighbours, as N addition increased the negative effects of neighbours on *L. virgaurea* and *T. mongolicum*, resulted in less biomass growth of them.

### The effect of habitat water condition on plant-plant interaction in response to N addition

N addition significantly reduced the negative neighbour effects on *K. macrantha* and *P. viviparum* at the wet site, but had no obvious effect on the plant-plant interactions at the dry site, showing that soil moisture altered the effect of N addition on plant-plant interactions. This provided a strong evidence to the theory that the outcomes of plant-plant interactions were dependent on multiple environmental variables^46^,^47^. According to the “multiple limitation hypothesis”^48^, plant growth is co-limited by multiple factors, and uptake of one nutrient depends on the availability of another nutrient. This may be helpful to understand the distinct results for the two sites. In contrast to the wet site where plant growth was limited mainly by N as in the common alpine grassland^20^,^22^, the dry site may also have been severely limited by water. Water stress can impair plant growth by reducing stomatal opening, limiting CO_2_ uptake, and reducing photosynthetic activity and plant N uptake^49^, thereby influencing plant response to N addition^50^. One possible reason that N addition did not reduce neighbour competition on *K. macrantha* and *P. viviparum* at the dry site may be that the two species did not benefit from increased N due to an actual water deficit. Moreover, as a result of excessive penetrability and drainage at the dry site, the added N may have been washed away by frequent precipitation and did not affect the possible “cascading effects” as much as in the wet site. Therefore, the growth of *K. macrantha* and *P. viviparum* without neighbours did not suffer from negative effects induced by increased N. The different effects of N addition on plant-plant interactions at the two sites demonstrated that habitat background could also be an important factor in affecting the response of plant-plant interactions to decreased stress, especially when the stress factor was seriously changed due to habitat variation.

### Implications for climate change adaptation of alpine grassland

The effects of plant life strategies and habitat water conditions on the responses of plant-plant interactions to increased N provided us with insight into the mechanisms of N addition controlling community structure and composition. Although plant community dynamics are simultaneously controlled by growth, reproduction and survival, the importance of clonal reproduction may be considerable for most species on the Tibetan Plateau^31^, and most of the dominant species are perennials^31^. Thus, the fitness of species on the Tibetan Plateau should largely depend on growth. The result showed that both the direct effects (effects on plant growth) and indirect effects (effects on competitive response ability) of N addition would benefit the growth, and thus fitness and dominance of N competitive species. N stress-tolerant species may not be negatively affected by N addition directly, while the fitness and dominance of these N stress-tolerant species would decrease due to enhanced neighbour competition on other resource like light and space. The different fates of plant growth induced by plant life strategies could be one of the main reasons for community structure changes in response to increased N due to N deposition, fertilization and accelerated mineralization on the Tibetan Plateau^26^,^32^.

Recent studies on exploring the effects of environmental changes on plant community structure found that the interaction between water fluctuation and N increase may be considerable^51^,^52^,^53^,^54^; however, few experiments tested the mechanism of the interaction effect on plant-plant interactions^55^. The result that negative neighbour effects on both of the N competitive species were not weakened by N addition at the dry site suggested that the positive effect of increased N on the growth, fitness and thus dominance of N competitive species might be weaker in some alpine habitats that are also severely limited by water. Bassin *et al*. also found that the positive effects of N addition on sedges (N limited) were much lower at a drier site in a sub-alpine grassland^50^. This indicated that the habitat water condition might change the effect of N addition on alpine plant community composition by substantially affecting the response of plant-plant interactions to increase in N. The number of species (i.e., two species) that we studied at the two moisture levels is not sufficient to answer the question of whether the effect of increased N on all species in alpine grassland will be weaker in a drier environment. However, the distinct difference between the wet and dry site provided us with an avenue such that we may deepen our knowledge of how fluctuating water levels and increased N interact by examining the effects of these factors on plant-plant interactions based on multiple species with different life strategies.

## Conclusion

Our results support the prediction that the effects of reduced stress on plant-plant interactions in alpine grassland depend on the plant life strategy of the targeted species. N addition intensified the negative effect of neighbours on species with a N stress-tolerant strategy and weakened the negative effect of neighbours on species with an N competitive strategy, but such an effect could be alleviated by reduced soil moisture. We believe that the different responses of alpine plants to increased N are one of the mechanisms driving the plant community changes on the Tibetan Plateau. Studies that take plant life strategy and the background of experiment site into consideration should be carried out to explain and predict the direct and indirect effects of climate change on alpine plant community.

## Methods

### Field Site

The experimental sites (33.26N, 102.22E) were established in an alpine meadow at 3570 m a.s.l. in the southeastern region of the Tibetan Plateau. The mean annual temperature is 1.6 °C with July and January averages of 11.1 and −10.1 °C, respectively, and the mean annual precipitation is approximately 786 mm with approximately 77.2% of the annual precipitation (~605 mm) distributed during the growing season (May to Sept.)^56^. The soil is classified as typical alpine Mat Cry-gelic Cambisols, with over 90% of the roots concentrated in the upper 15 cm of the soil^57^.

### Experimental design

We selected two sites with contrasting soil water conditions in a flat field. The selected sites were fenced and excluded from grazing since 2007. The relatively wet site was dominated by herbs such as *Deschampsia caespitosa, Elymus dahuricus,* and *Polygonum viviparum*. Other abundant species here were *Potentilla anserine, Kobresia macrantha, Sanguisorba officinalis, Taraxacum mongolicum* and *Ligularia virgaurea*. The mean (0–10 cm) soil water content in the growing season was 63.9% (hereafter wet site, i.e., the relatively wet site). The relatively dry site, created by artificial drainage in the late 1970s^58^, lying adjacent to the wet site, was co-dominated by herbs such as *Kobresia humilis*, *Potentilla discolor*, *Tibetia himalaica*, *Kobresia macrantha* and *Polygonum viviparum,* with a mean (0–10 cm) soil water content of 16.4% in the growing season (hereafter dry site, i.e., the relatively dry site). There were no obvious differences in physical background and management history between the two sites; therefore, the differences in vegetation and other soil indexes were attributed to the soil moisture changes induced by the drainage (Table 1).

*Kobresia macrantha* and *Polygonum viviparum* were commonly found at the two sites and were selected to examine the effect of moisture on the plant-plant interactions in response to N addition. Additionally, to test the influence of species life strategy, we chose another four species, i.e., *Sanguisorba officinalis*, *Potentilla anserine*, *Taraxacum mongolicum* and *Ligularia virgaurea*, at the wet site. All selected species are common perennial herbs on the Tibetan Plateau. Because it is hard to distinguish tillers from each other for *Deschampsia caespitosa* and *Elymus dahuricus*, we did not choose them as target species.

We carried out a neighbour removal experiment according to Callaway *et al*.^7^, with and without additional N, at both sites during the period from August 2012 to September 2014. In late August 2012, eight (2 m × 2 m) plots were randomly set up within a relatively homogenous area of the wet site and twelve same-sized plots were established at the dry site. Given that mature individuals may be more suitable to ensure a consistent initial state of the target species, we tagged the mature individuals immediately after these plots were selected. In each plot, we selected 1–4 pairs of individuals of each species with similar shoot size and leaf number. Then, 12–38 pairs of similar individuals of each species were tagged at each site (see Supplementary Table S5 for the number of pairs per species).

In mid May 2013, we randomly selected one individual of each pair, and clipped the aboveground biomass of neighbouring plants, from a radius of *c.* 15 cm around the selected individual. To prevent root competition from the surrounding vegetation, we cut the roots around the periphery of the removal circle. The neighbour effects on the target species were analysed by comparing the performance of target individuals without neighbours with that of controls (where neighbours were retained), as described by Klanderud^2^ and Callaway *et al*.^7^. To avoid interaction among tagged individuals, the paired individuals were located at least 30 cm away from each other, the individuals with retained neighbours were located at least 15 cm away from each other, and the individuals without neighbours were located at least 20 cm away from each other. After the neighbour removal, N fertilizer (NH_4_NO_3_, Chengdu Haihong Experimental Equipment Co., Ltd, Chengdu, China) was applied to one-half of the plots at each site at the rate of 10 g N m^−2^ yr^−1^ (this dose was proved to be the best for biomass accumulation in the area^26^,^59^). From then on, neighbour removal was conducted every two weeks. The number and length of the longest leaves (leaf number and leaf length, respectively) of all paired individuals were measured in late August. Then, the aboveground biomass of each individual was harvested and dried at 70°C in forced-air drying ovens (ZXFD-A5600, Hangzhou Chincan Trading Co., Ltd., Zhejiang, China) until constant weight. To test if there were any effects of year of experiment, the experiment was repeated from mid May 2014 to late August 2014.

To examine the effect of neighbour removal on the soil microenvironment, soil temperature was measured by DS1921G Thermocron iButton data loggers (Maxim Integrated Products, Inc., San Jose, CA 95134 USA). For each pair, we inserted the logger at 5-cm-depth soil in the neighbour area of both target individuals (about 5 cm away from targeted individuals), and recorded the values when stability was reached. We repeatedly measured both afternoon (14:00–15:00 hours) and pre-dawn (05:00–06:00 hours) soil temperature (17 June, 1 August and 17 August).

N utilization strategies of target species were evaluated by comparing the performances of them grown under neighbours retained condition in the control plots and the N addition plots. The effects of neighbour vegetation on performance of target species (i.e., response of target species to neighbour removal) were evaluated by comparing the performances of them grown with and without neighbours. We calculated a competition intensity index using the aboveground biomass, leaf length and leaf number data as original variables: log response ratio (LRR) = ln(Xc/Xr), where Xc and Xr are the values of the above variables in the presence and absence, respectively, of neighbours^1^,^60^. Positive LRR values indicate that the net interaction is facilitation, and negative values indicate competition.

### Statistical analyses

Soil temperature in the neighbour area of target individual was analysed by three-way repeated measures ANOVAs (RM-ANOVAs), with time as within-subject variable and experiment site (wet site vs. dry site) and removal of neighbour (Re, yes vs. no) as between-subject variables. Box M test was used to check the homogeneity of covariance matrices and the sphericity and compound symmetry in RM-ANOVAs.

To determine if N addition (increased vs. ambient), Re and year of experiment (Year, 2013 vs. 2014) had any impact on the vegetative growth of *K. macrantha*, *P. viviparum*, *S. officinalis*, *P. anserine*, *T. mongolicum* and *L. virgaurea* at wet site, we separately compared the biomass, leaf length and leaf number of individuals of each experimental treatment at the wet site using split-plot ANOVAs, where N addition, Year (main-plot factors), Re (subplot factors) and their interactions were considered as fixed factors, plot nested within all combinations of N addition and Year as a random factor, and LRR was considered as the dependent variable.

To test whether the effects of neighbour removal were affected by N addition and Year, we separately compared the biomass, leaf length and leaf number of individuals of each experimental treatment at the wet site using split-plot ANOVAs, where N addition, Year (main-plot factors), and their interactions were considered as fixed factors, plot nested within all combinations of N addition and Year were considered as a random factor, and LRR was considered as the dependent variable.

To determine whether there were any interaction effects between N addition, Year and experiment site with respect to the effects of neighbour removal, we compared LRR values for each index of *K. macrantha* and *P. viviparum* in each experimental treatment using split-plot ANOVAs with N addition, Year, experiment site (main-plot factors) and their interactions as fixed factors, plot nested within all combinations of N addition, Year and site as a random factor, and LRR was considered as the dependent variable.

Data were log transformed when they did not conform to the assumptions of normality and homogeneity of variances. All the statistical analyses were carried out using SPSS software (SPSS Inc., Chicago, USA), and all ANOVAs were conducted using the general linear models (GLM).

## Additional Information

**How to cite this article**: Wang, J. *et al*. Different responses of alpine plants to nitrogen addition: effects on plant-plant interactions. *Sci. Rep.* **6**, 38320; doi: 10.1038/srep38320 (2016).

**Publisher's note:** Springer Nature remains neutral with regard to jurisdictional claims in published maps and institutional affiliations.

## Supplementary Material

Supplementary Table

## Figures and Tables

**Table 1:** Dominant species, community height (cm), total vegetation coverage (%), average soil water content (%) of Dry site and Wet site.

Sites	Dominant species	Community height (cm)	Total vegetation coverage (%)	Average soil water content (%)
Dry site	*Kobresia humilis*, *Potentilla discolor*, *Tibetia himalaica*, *Kobresia macrantha*	12.7 ± 1.9	73.6 ± 3.3	16.4 ± 3.5
Wet site	*Deschampsia caespitosa*, *Elymus dahuricus*, *Polygonum viviparum*	26.1 ± 1.4	98.1 ± 1.1	63.9 ± 5.5

(mean ± SD).
