# Supplementary material for: Different responses of alpine plants to nitrogen addition: effects on plant-plant interactions
Source: Sci Rep. 2016 Dec 6;6:38320. doi: 10.1038/srep38320 (PMC5138851; doi:10.1038/srep38320)
Supplement: Supplementary Table [file srep38320-s1.pdf]

Supplementary Info

Different responses of alpine plants to nitrogen addition: effects on plant-plant interactions

Jun Wang [1, 2,3], Peng Luo [1,*], Hao Yang [1, 2,3], Chengxiang Mou [1,3], Li Mo [1,3]

[1]Key Laboratory of Mountain Ecological Restoration and Bioresource Utilization of Chinese Academy of Sciences, Chengdu Institute of Biology, Chinese Academy of Sciences, Chengdu, 610041, China

[2] College of Life Science, Sichuan University, Chengdu, 610064, China

[3] University of Chinese Academy of Sciences, Beijing, 100000, China

[*] Corresponding author: Luopeng@cib.ac.cn

Table S1. Repeated measures ANOVA tables (significant at < 0.05 in bold) for the effects of Re = removal of neighbor (yes vs. no), Site = experiment site (Wet site vs. Dry site) and their interactions on soil temperature (5-cm depth) in the neighbour areas of target individuals (about 5 cm away from targeted individuals).

Sources of Variation	Pre-dawn		Afternoon	
	$F_{d.f.}$	P	$F_{d.f.}$	P
Site	$0.597_{1,8}$	**0.462**	$2509.34_{1,8}$	**<0.001**
Re	$31.61_{1,8}$	**<0.001**	$316.40_{1,8}$	**<0.001**
Re*Site	$9.958_{1,8}$	**0.013**	$5.536_{1,8}$	**0.046**

Table S2. Split-plot ANOVA tables (significant at < 0.05 in bold) for the effects of Year = year of experiment (2013 vs. 2014), N = nutrient addition (increased vs. ambient, main-plot factors), Re = removal of neighbor (yes vs. no, subplot factor), their interactions and plot nested within all combination of Year and N (random factor) on number of leaves (LN), length of the longest leaf (LL) and abovegound biomass (AB) of all target species in Wet site.

Species	Indexes	Year		N		Re		N*Re		Year*N		Year*Re		Year*Re*N		Plot (N*Year)	
		$F_{d.f.}$	*P*	$F_{d.f.}$	*P*	$F_{d.f.}$	*P*	$F_{d.f.}$	*P*	$F_{d.f.}$	*P*	$F_{d.f.}$	*P*	$F_{d.f.}$	*P*	$F_{d.f.}$	*P*
K. macrantha	LN	$2.43_{1,12}$	0.143	$0.07_{1,12}$	0.793	$89.43_{1, 78}$	**<0.001**	$7.81_{1, 78}$	**0.007**	$0_{1,12}$	0.989	$0.36_{1, 78}$	0.553	$0.2_{1, 78}$	0.656	$1.37_{12,78}$	0.199
	LL	$0.13_{1,12}$	0.726	$0.02_{1,12}$	0.893	$232.51_{1, 78}$	**<0.001**	$41.49_{1, 78}$	**<0.001**	$0.64_{1,12}$	0.439	$3.31_{1, 78}$	0.073	$0.69_{1, 78}$	0.408	$1.11_{12,78}$	0.362
	AB	$0.29_{1,12}$	0.598	$0.17_{1,12}$	0.686	$0.27_{1, 78}$	0.603	$62.8_{1, 78}$	**<0.001**	$0.04_{1,12}$	0.85	$1.52_{1, 78}$	0.222	$0.21_{1, 78}$	0.651	$3.22_{12,78}$	**0.001**
P. viviparum	LN	$0.04_{1,12}$	0.853	$53.83_{1,12}$	**<0.001**	$39.15_{1,52}$	**<0.001**	$21.73_{1,52}$	**<0.001**	$0.63_{1,12}$	0.442	$0.08_{1,52}$	0.785	$0.04_{1,52}$	0.834	$1.25_{12,52}$	0.279
	LL	$0.39_{1,12}$	0.543	$8.32_{1,12}$	**0.013**	$207.93_{1,52}$	**<0.001**	$23.79_{1,52}$	**<0.001**	$0.01_{1,12}$	0.937	$0_{1,52}$	0.986	$0.01_{1,52}$	0.937	$2.37_{12,52}$	**0.016**
	AB	$0.87_{1,12}$	0.368	$3.58_{1,12}$	0.083	$3.32_{1,52}$	0.074	$30.41_{1,52}$	**<0.001**	$0.01_{1,12}$	0.928	$0.4_{1,52}$	0.529	$0.35_{1,52}$	0.554	$0.52_{12,52}$	0.147
S. officinalis	LN	$0.01_{1,12}$	0.938	$1.42_{1,12}$	0.256	$203.83_{1,72}$	**<0.001**	$3.28_{1,72}$	0.074	$0.33_{1,12}$	0.578	$0.09_{1,72}$	0.76	$0.19_{1,72}$	0.66	$3.58_{12,72}$	**<0.001**
	LL	$3.26_{1,12}$	0.095	$3.2_{1,12}$	0.098	$220.71_{1,72}$	**<0.001**	$3.24_{1,72}$	0.076	$0.01_{1,12}$	0.942	$1.59_{1,72}$	0.212	$0.89_{1,72}$	0.348	$2.17_{12,72}$	**0.022**
	AB	$1.08_{1,12}$	0.317	$0.65_{1,12}$	0.435	$41.83_{1,72}$	**<0.001**	$0.4_{1,72}$	0.53	$0.24_{1,12}$	0.632	$2.19_{1,72}$	0.413	$0.21_{1,72}$	0.648	$1.69_{12,72}$	**0.091**
P. anserine	LN	$1.33_{1,12}$	0.268	$1.89_{1,12}$	0.198	$192.6_{1,94}$	**<0.001**	$6.09_{1,94}$	**0.015**	$0.01_{1,12}$	0.908	$0.28_{1,94}$	0.596	$0.02_{1,94}$	0.898	$1.33_{12,94}$	0.215
	LL	$7.18_{1,12}$	**0.017**	$37.18_{1,12}$	**<0.001**	$231.41_{1,94}$	**<0.001**	$5.63_{1,94}$	**0.02**	$0.37_{1,12}$	0.555	$2.7_{1,94}$	0.104	$0_{1,94}$	0.982	$0.67_{12,94}$	0.774
	AB	$0.08_{1,12}$	**0.784**	$0.34_{1,12}$	**0.496**	$235.04_{1,94}$	**<0.001**	$4.76_{1,94}$	**0.032**	$0.86_{1,12}$	0.369	$2.97_{1,94}$	0.088	$0.64_{1,94}$	0.426	$0.84_{12,94}$	0.611
T. mongolicum	LN	$0.63_{1,12}$	0.443	$0.5_{1,12}$	0.495	$558.41_{1,94}$	**<0.001**	$4.76_{1,94}$	**0.032**	$0.06_{1,12}$	0.806	$0.4_{1,94}$	0.531	$0.04_{1,94}$	0.844	$2.37_{12,94}$	**0.01**
	LL	$32.62_{1,12}$	**<0.001**	$0.05_{1,12}$	0.824	$210.29_{1,94}$	**<0.001**	$0.36_{1,94}$	0.548	$1.21_{1,12}$	0.292	$0.52_{1,94}$	0.472	$3.4_{1,94}$	0.068	$1.13_{12,94}$	0.35
	AB	$2.09_{1,12}$	0.173	$1.82_{1,12}$	0.201	$254.47_{1,94}$	**<0.001**	$4_{1,94}$	**0.048**	$0.05_{1,12}$	0.828	$7.61_{1,94}$	**0.007**	$0_{1,94}$	0.966	$2_{12,94}$	**0.032**
L. virgaurea	LN	$0.24_{1,12}$	0.628	$2.96_{1,12}$	0.1	$113.72_{1,52}$	**<0.001**	$13.04_{1,52}$	**0.001**	$0_{1,12}$	1	$0.85_{1,52}$	0.359	$0.01_{1,52}$	0.933	$0.93_{12,52}$	0.521
	LL	$0.44_{1,12}$	0.515	$9.45_{1,12}$	**0.006**	$16.22_{1,52}$	**<0.001**	$35.11_{1,52}$	**<0.001**	$0.01_{1,12}$	0.931	$0.01_{1,52}$	0.905	$0.15_{1,52}$	0.696	$0.84_{12,52}$	0.613
	AB	$1.09_{1,12}$	0.304	$58.55_{1,12}$	**<0.001**	$96.81_{1,52}$	**<0.001**	$30.38_{1,52}$	**<0.001**	$0.07_{1,12}$	0.792	$0.09_{1,52}$	0.673	$0.08_{1,52}$	0.781	$0.44_{12,52}$	0.942

Table S3. Split-plot ANOVA tables (significant at < 0.05 in bold) for the effects of Year = year of experiment (2013 vs. 2014), N = nutrient addition (increased vs. ambient, main-plot factors), their interactions and plot nested within all combination of Year and N (random factor) on log response ratio of number of leaves (LRR(LN)), length of the longest leaf (LRR(LL)) and abovegound biomass (LRR(AB) of all species in Wet site.

Species	Indexes	Year		N		Year*N		Plot (Year*N)	
		$F_{\text{d.f.}}$	*P*	$F_{\text{d.f.}}$	*P*	$F_{\text{d.f.}}$	*P*	$F_{\text{d.f.}}$	*P*
K. macrantha	LRR(LN)	$0.73_{1,12}$	0.407	$10.7_{1,12}$	**0.007**	$0.05_{1, 12}$	0.833	$1.95_{12, 33}$	0.064
	LRR(LL)	$5.6_{1,12}$	**0.032**	$55.98_{1,12}$	**<0.001**	$2.23_{1, 12}$	0.156	$0.52_{12, 33}$	0.883
	LRR(AB)	$8.5_{1,12}$	**0.01**	$284.29_{1,12}$	**<0.001**	$0.96_{1, 12}$	0.341	$0.37_{12, 33}$	0.964
P. viviparum	LRR(LN)	$1.27_{1,12}$	0.28	$11.7_{1,12}$	**0.004**	$0.08_{1,20}$	0.777	$1.16_{12,20}$	0.369
	LRR(LL)	$0_{1,12}$	0.97	$14.47_{1,12}$	**0.002**	$0_{1,20}$	0.97	$1.92_{12,20}$	0.095
	LRR(AB)	$1.4_{1,12}$	0.255	$133.75_{1,12}$	**<0.001**	$1.48_{1,20}$	0.244	$0.63_{12,20}$	0.795
S. officinalis	LRR(LN)	$0.27_{1,12}$	0.612	$1.81_{1,12}$	0.198	$0.1_{1,36}$	0.759	$1.15_{12,36}$	0.352
	LRR(LL)	$0.8_{1,12}$	0.387	$3.49_{1,12}$	0.082	$0.65_{1,36}$	0.432	$1.37_{12,36}$	0.224
	LRR(AB)	$2.16_{1,12}$	0.163	$0.05_{1,12}$	0.821	$1.45_{1,36}$	0.248	$1.34_{12,36}$	0.24
P. anserine	LRR(LN)	$0.15_{1,12}$	0.704	$1.84_{1,12}$	0.198	$0.02_{1,41}$	0.897	$3.63_{12,41}$	**0.001**
	LRR(LL)	$15.81_{1,12}$	**0.001**	$7.71_{1,12}$	**0.014**	$0.02_{1,41}$	0.896	$0.66_{12,41}$	0.78
	LRR(AB)	$6.38_{1,12}$	**0.025**	$6.45_{1,12}$	**0.025**	$2.15_{1,41}$	0.163	$1.7_{12,41}$	0.103
T. mongolicum	LRR(LN)	$1.09_{1,12}$	0.317	$4.95_{1,12}$	**0.045**	$0.22_{1,41}$	0.65	$4.14_{12,41}$	**<0.001**
	LRR(LL)	$2.22_{1,12}$	0.161	$0.39_{1,12}$	0.541	$2.2_{1,41}$	0.162	$1.67_{12,41}$	0.109
	LRR(AB)	$5.36_{1,12}$	0.039	$4.82_{1,12}$	**0.048**	$0_{1,41}$	0.969	$3.39_{12,41}$	**0.002**
L. virgaurea	LRR(LN)	$0.33_{1,12}$	0.574	$13.58_{1,12}$	**0.002**	$0_{1,24}$	0.975	$1.05_{12,24}$	0.443
	LRR(LL)	$0.69_{1,12}$	0.414	$80.65_{1,12}$	**<0.001**	$0.09_{1,24}$	0.765	$0.48_{12,24}$	0.908
	LRR(AB)	$0.01_{1,12}$	0.926	$20.27_{1,12}$	**<0.001**	$0.06_{1,24}$	0.808	$0.56_{12,24}$	0.849

Table S4. Split-plot ANOVA tables (significant at < 0.05 in bold) for the effects of Year = year of experiment (2013 vs. 2014), N = nutrient addition (increased vs. ambient), Site = experiment site (wet site vs. dry site, main-plot factors), their interactions and plot nested within all combination of Year, N and Site (random factor) on log response ratio of number of leaves (LRR(LN)), length of the longest leaf (LRR(LL)) and abovegound biomass (LRR(AB) of *K. macrantha* and *P. viviparum*.

Sources of Variation	LRR(LN)		LRR(LL)		LRR(AB)	
	$F_{d.f.}$	*P*	$F_{d.f.}$	*P*	$F_{d.f.}$	*P*
K. macrantha						
Year	$1_{1,20}$	0.324	$6.57_{1,20}$	**0.014**	$8.48_{1,20}$	**0.006**
N	$7.2_{1,20}$	**0.011**	$44.51_{1,20}$	**<0.001**	$67.42_{1,20}$	**<0.001**
Site	$7.86_{1,20}$	**0.008**	$33.31_{1,20}$	**<0.001**	$19.43_{1,20}$	**<0.001**
Year*N	$0.51_{1,20}$	0.48	$2.37_{1,20}$	0.132	$2.1_{1,20}$	0.156
Year*Site	$0.42_{16,38}$	0.523	$2.15_{16,38}$	0.151	$0.04_{16,38}$	0.838
N*Site	$12.81_{1,20}$	**0.001**	$36.58_{1,20}$	**<0.001**	$53.42_{1,20}$	**<0.001**
Year*N*Site	$0.08_{16,38}$	0.778	$0.97_{16,38}$	0.33	$0.27_{16,38}$	0.609
Plot(Year * N * Site)	$1_{16,38}$	0.479	$0.5_{16,38}$	0.985	$0.76_{16,38}$	0.804
P. viviparum						
Year	$0.08_{1,20}$	0.777	$2.85_{1,20}$	**0.1**	$0.99_{1,20}$	0.325
N	$1.5_{1,20}$	0.229	$14.08_{1,20}$	**0.001**	$56.85_{1,20}$	**<0.001**
Site	$0.18_{1,20}$	0.674	$78.07_{1,20}$	**0.004**	$65.72_{1,20}$	**<0.001**
Year*N	$1.48_{1,20}$	0.231	$0_{1,20}$	0.982	$0.01_{1,20}$	0.943
Year*Site	$0.86_{16,38}$	0.359	$2.72_{16,38}$	0.108	$0.31_{16,38}$	0.58
N*Site	$14.81_{1,20}$	**<0.001**	$5.54_{1,20}$	**0.024**	$57.97_{1,20}$	**<0.001**
Year*N*Site	$1.01_{16,38}$	0.332	$0.14_{16,38}$	0.714	$2.18_{16,38}$	0.148
Plot(Year * N * Site)	$1.31_{16,38}$	0.197	$1.4_{16,38}$	0.145	$0.8_{16,38}$	0.741

Table S5. Number of pairs selected for each targeted species under nature condition (CK) and additional N (N) in 2013 and 2014, respectively.

Site and species	2013		2014	
	CK	N	CK	N
**Wet site**				
K. macrantha	9	11	13	16
P. viviparum	8	7	10	11
S. officinalis	13	9	11	13
P. anserine	19	11	11	16
T. mongolicum	16	10	16	15
L. virgaurea	6	6	12	16
**Dry site**				
K. macrantha	16	22	14	20
P. viviparum	14	9	16	12
